# Biologically Active Compounds Present in Tobacco Smoke: Potential Interactions Between Smoking and Mental Health

**DOI:** 10.3389/fnins.2022.885489

**Published:** 2022-04-26

**Authors:** Sa Weon Hong, Paul Teesdale-Spittle, Rachel Page, Bart Ellenbroek, Penelope Truman

**Affiliations:** ^1^School of Health Sciences, Massey University, Wellington, New Zealand; ^2^School of Biological Sciences, Victoria University of Wellington, Wellington, New Zealand; ^3^Department of Psychology, Victoria University of Wellington, Wellington, New Zealand

**Keywords:** tobacco dependence, nicotine, tobacco smoke components, monoamine oxidase inhibition, mental health, Parkinson’s Disease

## Abstract

Tobacco dependence remains one of the major preventable causes of premature morbidity and mortality worldwide. There are well over 8,000 compounds present in tobacco and tobacco smoke, but we do not know what effect, if any, many of them have on smokers. Major interest has been on nicotine, as well as on toxic and carcinogenic effects and several major and minor components of tobacco smoke responsible for the negative health effects of smoking have been elucidated. Smokers themselves report a variety of positive effects from smoking, including effects on depression, anxiety and mental acuity. Smoking has also been shown to have protective effects in Parkinson’s Disease. Are the subjective reports of a positive effect of smoking due to nicotine, of some other components of tobacco smoke, or are they a manifestation of the relief from nicotine withdrawal symptoms that smoking provides? This mini-review summarises what is currently known about the components of tobacco smoke with potential to have positive effects on smokers.

## Introduction

Smoking is a major cause of preventable premature death and disability, believed to cause six million deaths worldwide each year with smokers, on average, losing 10 years of their lives (West, 2017). The negative health effects of prolonged smoking are well established. Smoking primarily damages lung and cardiovascular health, as well as impacting negatively on every organ of the body (U.S. Department of Health and Human Services., 2004). The reason people keep smoking, despite knowing that this habit is likely to eventually kill them, is that smoking is addictive (Henningfield and Fant, 1999). The major addictive component of tobacco smoke is nicotine, but it is increasingly evident that tobacco dependence is multi-faceted (West and Cox, 2021). Nicotine in laboratory tests is much less addictive than the lived experience of smokers would suggest (Jain, 2003; Balfour, 2009). The explanations are varied, ranging from societal and behavioural influences (Moolchan et al., 2003), to strong cue association and cognitive effects (Sacco et al., 2004; Chiamulera, 2005) and to the influence of tobacco companies (Hoek et al., 2012).

Smokers themselves report smoking relieves stress and anxiety, and aids concentration (Benowitz, 2010) and smokers with a variety of mental health conditions report using smoking as a form of self-medication (Leonard et al., 2001; Aubin et al., 2012). Unsurprisingly, the interpretation of these reports varies widely. However, smoking is proven to have protective effects in Parkinson’s Disease (Castagnoli and Murugesan, 2004; Gigante et al., 2017), with smoking having a well-established neuroprotective effect against this disease (Veljkovic et al., 2018).

A strong theme coming through the literature is that components in tobacco smoke other than nicotine, possibly monoamine oxidase (MAO) inhibitors, may enhance tobacco dependence, or may have positive effects on mood (Fowler et al., 2003; Rose, 2006; Hogg, 2016; Harris et al., 2020). Thus, apart from the effects of nicotine, the observed difficulty that people have in stopping smoking could be influenced by other chemical components of the tobacco smoke. Such interpretations are controversial. For instance, the high rate of relapse in smokers attempting to stop smoking is attributed to relief from nicotine withdrawal, rather than thinking of a positive effect on mood or concentration from smoking as reinforcing nicotine dependence (Moylan et al., 2013).

It is completely accepted that tobacco smoking is a harmful habit, due to the many toxic and carcinogenic components of the smoke. However, this review aims to focus attention on areas of the literature suggesting pharmacological drivers behind tobacco dependence other than the immediate effects of nicotine in inducing dependence, and our current understanding of the short-term effects of tobacco smoke components on smokers. Are some of these effects positive, as in neuroprotection against Parkinson’s Disease? Are there known tobacco components which could have a positive effect?

## Nicotine

Nicotine acts at the nicotinic acetylcholine receptors (nAChRs) to cause flow-on effects in specific areas of the brain. These nAChRs are believed to be important in coordinating brain responses, by action of the natural transmitter, acetylcholine (ACh). Nicotine binds to nAChRs mimicking the action of ACh and altering responses within the brain (Balfour, 2009).

The key addictive response is stimulation of nAChRs in the ventral tegmental area of the brain, which causes the release of dopamine in the nucleus accumbens, believed to be central to the development of all addictive responses (Di Chiara and Imperato, 1988). Nicotine’s pharmacology has been well reviewed by others (Benowitz, 1996; Laviolette and van der Kooy, 2004) as have the complexities of the nicotinic receptors in the brain (Albuquerque et al., 2009; Wu and Lukas, 2011). The nAChRs belong to the ionotropic family of receptors, with five proteins forming a channel. Seventeen different proteins have been identified, leading to a wide diversity of nicotinic receptor subtypes. Thus the diversity of nicotinic receptor types and their varied localisation within the brain allows for much more nuanced and complex responses to nicotine than simple dopamine release.

Several milligrams of nicotine are present in each gram of tobacco, and nicotine reaches around 0.2 micromolar concentrations in the blood of smokers, sufficient to cause nAChR responses (Alkondon et al., 2000). Nicotine concentrations rise rapidly when tobacco smoke is inhaled, reaching the brain in under two minutes. It then dissipates slowly, having a half-life of around 2 h (Benowitz, 2009). As nicotine brain concentrations fall, in the addicted smoker, cravings for nicotine begin, leading to smokers repeating the experience. Many of the effects of smoking tobacco (dopamine release and dependence, and withdrawal effects and relapse back to smoking) can be related back to these key effects of nicotine on the brain.

As the major pharmacologically active component of tobacco smoke, nicotine has also been investigated to see whether it can cause other effects, reported from smoking, such as relief from anxiety and depression, improved concentration and symptom control in Schizophrenia, Parkinson’s Disease, Attention Deficit Hyperactivity Disorder, and Alzheimer’s Disease (Mihailescu and Drucker-Colin, 2000; Newhouse et al., 2004a; Veljkovic et al., 2018).

### Nicotine and Cognition/Concentration

*In utero* exposure and exposure of children to tobacco smoke are both believed to interfere with cognitive development, causing deleterious effects on attention span and ability to concentrate, in children (Alhowail, 2021; Hajdusianek et al., 2021). With some caveats as to the strength of the evidence (Chan et al., 2020) it is generally accepted that smoking in pregnancy is bad for the unborn child, although there is some doubt as to whether nicotine causes all of the problems noted (Baler et al., 2008; Chan et al., 2020).

In adults, however, nicotine is believed to have a positive effect on mental acuity (Conley et al., 2021; Nop et al., 2021). Again, this has been suggested by studies in animals and in humans (Kumari et al., 2003; Newhouse et al., 2004b), and trials of the effect of nicotine in older adults have produced some evidence of benefit. A recent meta-analysis by Majdi and coworkers suggested that nicotine has a moderate but positive effect on attentional ability in healthy non-smoking adults (Majdi et al., 2021) and it has been suggested that nicotine could be used to treat late-life depression, with part of this action being mediated by effects on cognition (Gandelman et al., 2018).

Smoking as a long-term enhancer of cognition is not recommended, however, since smoking is a risk factor for vascular dementia (Lopez-Arrieta et al., 2001) as well as for many other health conditions.

### Nicotine and Anxiety/Depression

The suggestion that nicotine can relieve stress and help with anxiety and depression is controversial. The key argument is whether the positive effects noted by smokers are real and act as a reinforcer of tobacco dependence by improving mood (Pomerleau et al., 1984; Choi et al., 2015) or whether nicotine dependence causes depression and/or anxiety over time, with relief of cravings being misread by smokers as relief from the related mood disorders (Moylan et al., 2013; Molas et al., 2017). A systematic review by Fluharty and co-workers (Fluharty et al., 2017) found evidence for causation in both directions and suggested the need for more studies. More recent work tends to look at effects of transdermal nicotine, and has found potential for nicotine to be used in cases of major depressive disorder (Janes et al., 2018), and for relief of later life depression (Conley et al., 2021).

### Nicotine and Schizophrenia

Schizophrenia is also strongly associated with tobacco smoking, however, the direction of causation is controversial. Scott and coworkers examined the evidence that smoking might cause schizophrenia and suggested nicotine as the causative agent (Scott et al., 2018). Others have suggested that nicotine’s effects within the brain might give relief from the symptoms of schizophrenia, providing motivation to continue smoking (Postma et al., 2006). It is possible that nicotine’s positive effect on cognition is the mediating mechanism for this (Waterhouse et al., 2018).

### Nicotine and Alzheimer’s Disease

The possibility that nicotine might be useful in treating early stages of Alzheimer’s Disease has been studied for some years. As with Parkinson’s Disease, smokers are under-represented among those with Alzheimer’s Disease. Impaired cholinergic function is an early feature of Alzheimer’s Disease and nAChR agonists have been suggested as potentially helpful in ameliorating symptoms (Albuquerque et al., 2001). Transdermal nicotine has been used in trials with positive effects on attention and other cognitive measures (Newhouse et al., 2004b). Nicotine has also been shown to be effective in reducing behavioural and synaptic plasticity deficits in a rat model of Alzheimer’s disease (Esteves et al., 2017). In a separate line of enquiry nicotine is also thought to delay formation of amyloid plaque (Zhang et al., 2006), giving another mechanism by which smoking (nicotine) could be helpful in delaying Alzheimer’s Disease onset.

### Nicotine and Parkinson’s Disease

While smoking is well known to be protective against Parkinson’s Disease (Gigante et al., 2017), the causative agents in this case are likely to include both nicotine, though its action on dopaminergic pathways (Thiriez et al., 2011), and monoamine oxidase inhibitors (Castagnoli and Murugesan, 2004). Additionally, Kardani et al. (2017) have demonstrated that nicotine can slow the formation of α-synuclein fibrils. However, although nicotine appeared to be neuroprotective in Parkinsonian animals it had no effect in restoring damage in the same experimental system (Huang et al., 2009) and was not significantly effective in Phase II clinical trials (Villafane et al., 2018).

## Minor Tobacco Alkaloids

Like nicotine, the other nicotine analogues found in tobacco smoke have also been found to act on the nicotinic receptors. The “minor alkaloids” in tobacco smoke other than nicotine include nornicotine, myosmine, cotinine, anabasine and anatabine. Where nicotine is found at around 10 milligrams per cigarette in tobacco, these minor alkaloids are found in microgram per cigarette amounts (Smith et al., 2015).

Several groups have looked at the effect of these alkaloids *in vivo* (Harris et al., 2015; Marusich et al., 2017; Tan et al., 2022). Such studies have found that the minor tobacco alkaloids can partially substitute for nicotine in behavioural tests but have lower potency than nicotine itself. The different alkaloids are not equivalent in their behavioural effects. Differences in binding to different nAChR variants between the various nicotinic alkaloids would allow for differences in mode of action and potency even if all act through binding nAChRs. The combination of lower potency and lower concentration in tobacco make it unlikely that these alkaloids have significant effects on smoker behaviour.

Cotinine, a breakdown product of nicotine, has relatively long half-life in the blood [c.a. 16 h compared to c.a. 2 h for nicotine (Hukkanen et al., 2005)] and reaches low micromolar concentrations in a smoker’s blood, several-fold higher than the concentration of nicotine. It may, therefore, be present in sufficient amounts to have a modulating effect on smoker behaviour, particularly for heavy smokers. Cotinine is a weak agonist of nAChRs, binding less strongly than nicotine (Tan et al., 2021) but has recently been shown to support self-administrative behaviour (Tan et al., 2022) although less robustly than nicotine. Cotinine upregulates the α7nAChRs and this activity is neuroprotective to glial cells (Iarkov et al., 2021). Cotinine also slows down the formation of α-synuclein fibrils, with a potency similar to that of nicotine, and may also have positive cognitive benefits.

Table 1 summarises the suggested beneficial effects of nicotine and cotinine.

**TABLE 1:** Nicotine, cotinine, and monoamine oxidase inhibitors in tobacco and tobacco smoke: potential positive effects.

Smoke component	Chemical structure	Mechanism	Disease state affected	References
Nicotine		nAChR activation	Cognitive improvement	Newhouse et al., 2004b; Majdi et al., 2021
Nicotine	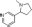	↓α-synuclein fibril formation: (cognition ↑)	Parkinson’s disease	Kardani et al., 2017; Villafane et al., 2018
Nicotine		↓Amyloid ß-peptide aggregation: (cognition↑)	Alzheimer’s disease	Zhang et al., 2006; Esteves et al., 2017
Nicotine		Cognition↑	Schizophrenia	Waterhouse et al., 2018
Nicotine		Cognition ↑	Depression	Gandelman et al., 2018; Conley et al., 2021

Cotinine	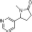	↓α-synuclein fibril formation, neuroprotection	Parkinson’s disease Alzheimer’s disease	Terry et al., 2005; Riveles et al., 2008; Iarkov et al., 2021;

Naphthoquinones	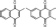	MAO inhibition, neuroprotection (nitric oxide control)	Parkinson’s disease	Venkatakrishnan et al., 2009

Harman/Norharman	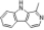	MAO inhibition	Antidepressant	Farzin and Mansouri, 2006
Harman/Norharman	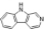	MAO inhibition	Antianxiolytic	Smith et al., 2013

2,3,6-Trimethyl-1,4-naphthoquinone	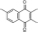	MAO inhibition	Neuroprotection, Parkinson’s disease	Sari and Khalil, 2015

## Monoamine Oxidase Inhibitors

Monoamine oxidase inhibitors (MAOIs) in tobacco smoke have long been regarded as having potential significance in modulating the effects of smoking on the brain (Fowler et al., 2003; Dome et al., 2010; Hogg, 2016). Monoamine oxidase (MAO) enzymes in the brain are responsible for clearance of brain transmitters, notably dopamine, serotonin, adrenaline and noradrenaline (Lewis et al., 2007). Since inhibition of MAO activity will lead to reduced clearance of neurotransmitters such as dopamine it is suggested that MAOIs in the tobacco smoke might enhance the dopamine reward from nicotine and the addictiveness of smoking.

A variety of experimental evidence has been produced showing that MAO inhibition enhances behavioural responses to nicotine in rats (Guillem et al., 2005; Villegier et al., 2006, 2011; Smith et al., 2016b; Harris et al., 2020) but the extension to human smoking behaviour is less clear. MAO activity is well known to be reduced in smokers (Fowler et al., 1996a,b) with the inhibition being believed to be irreversible (Yu and Boulton, 1987). The time course of recovery of activity after smoking cessation extends over several weeks after smoking cessation, consistent with the equivalent recovery time from inhibition by known irreversible MAO inhibitory drugs (Fowler et al., 2003). Although epigenetic (Launay et al., 2009) and microRNA (Higuchi et al., 2018) mechanisms for MAO activity reduction have been suggested, direct inhibition by components of tobacco smoke as the major cause of the observed reduction remains a likely mechanism by which MAO inhibition is accomplished in smokers.

The known MAOIs in tobacco smoke include the ß-carbolines harman and norharman, α-naphthylamine, farnesyl acetone and tetrahydroisoquinolines (TIQ’s) (Lewis et al., 2007; Hogg, 2016). Harman and norharman have been proposed as the major contributors to the observed MAOI activity in tobacco smoke, causing the observed decrease in MAO activity (Rommelspacher et al., 2002; Herraiz and Chaparro, 2005), with the discrepancy between the amounts of ß-carboline measured in smokers, and the inhibition observed being ascribed to their accumulation in platelets (Rommelspacher et al., 2002) and in brain (Fekkes and Bode, 1993). However, harman and norharman make up less than 1/10th of the total direct MAO inhibitory activity in tobacco smoke (Truman et al., 2017) so the opportunity for other MAOIs in tobacco smoke to contribute substantially to the MAO activity reduction seen in smokers must exist. No irreversible MAO inhibitors have yet been reported from tobacco or tobacco smoke.

The question of whether MAO inhibitors in tobacco smoke can affect behaviour remains unresolved. As well as the potential for effects on addiction, MAO enzymes are drug targets for a variety of neurological disorders including depression, mood, anxiety, attention deficit hyperactivity, Tourette’s syndrome, Parkinson’s disease and Alzheimer’s disease (Sharama, 2016; Borroni et al., 2017). Of the known MAO inhibitors in tobacco smoke, high concentrations of harman and norharman can affect responses to nicotine (Harris et al., 2020) and may act as antidepressants (Farzin and Mansouri, 2006; Smith et al., 2013) in animals. However, when used in amounts relevant to smokers, they were not seen to affect rat self-administration of nicotine (Smith et al., 2015) and no pharmacological effects have been reported from the known tobacco MAO inhibitors at physiologically relevant concentrations. In contrast, use of tobacco smoke extracts in self-administration or intracranial self-stimulation experiments has been found to affect responses to nicotine (Harris et al., 2010; Costello et al., 2014; Brennan et al., 2015). This discrepancy may be in part because the full range of tobacco smoke MAO inhibitors has not yet been identified.

It has also been suggested that further MAO inhibitory activity is formed from smoke components in the body. Acetaldehyde is formed during tobacco combustion, from the sugars in the plant material, and from sugars added as humectants and flavour additives during tobacco and cigarette manufacture. Acetaldehyde is typically present in cigarette smoke amounts ranging from 0.6 to over 2 milligrams per cigarette (Seeman et al., 2002). Acetaldehyde yields locomotor stimulation and reinforcing effects (Quertemont and Tambour, 2004) at concentrations higher than those seen in tobacco smoke and enhances self-administration of nicotine at concentrations similar to those in tobacco smoke (Belluzzi et al., 2005), although this finding was not confirmed by Smith and co-workers in similar trials (Smith et al., 2015).

It is suggested that acetaldehyde acts by reacting with chemicals naturally occurring in the brain to form MAO inhibitors (Talhout et al., 2007). A wide variety of TIQs are formed from acetaldehyde and catecholamines (dopamine, noradrenalin, adrenalin) (Naoi et al., 2004; Patsenka and Antkiewicz-Michaluk, 2004). Of particular note are the cyano derivatives, which inhibit MAO-A and -B with Ki values between 18 and 38 μM (Mendez-Alvarez et al., 1997). A group of tetrahydro-ß-carbolines (THBCs) are also formed from condensation of acetaldehyde and indoleamines (serotonin, tryptamine, tryptophan) (Talhout et al., 2007) and are more potent, inhibiting with Ki values under 10 μM. It seems likely that concentrations of these MAOIs, formed in the body after smoking, are sufficient to have some effect on smoking addiction in humans, as well as in rats, since a randomized double-blind trial of a method to reduce the amount of acetaldehyde entering a smoker’s body had some success in encouraging cessation (Syrjanen et al., 2017). Thus far, together with the ß-carbolines, acetaldehyde is a leading candidate as a tobacco smoke component causing monoamine oxidase inhibition in smokers sufficient to modulate tobacco dependence, even though it does not directly cause MAO inhibition. The inhibitors formed from acetaldehyde appear to be reversible (Naoi et al., 2004).

The immediate effect on MAO activity of smoking a single cigarette could not be detected using PET methods (Fowler et al., 2003), whereas longer term effects of continued smoking results in an overall, apparently irreversible reduction of 30–40% in both MAO-A and -B activity, suggesting that long term exposure to tobacco smoke is required for this effect. Until we know which components of tobacco smoke cause the observed inhibition of MAO activity, and their mechanism of action, it will remain difficult to determine the extent, timing and overall effects of MAO inhibition in smokers.

Monoamine oxidase inhibitors in tobacco smoke may well have effects other than enhancing the addictive potential of nicotine. Smith and coworkers (Smith et al., 2016a) have examined the effects of MAO inhibitors and nicotine on brain function, measured using EEG techniques in non-smoking humans. They suggest that MAO inhibition alters brain function leading to lapses in cognition. Nicotine’s effect in enhancing cognition is suggested to alleviate this.

Monoamine oxidase inhibitors are a major drug target, of interest for treatment of depression and anxiety, Parkinson’s disease and Alzheimer’s disease (Sharama, 2016). It has been suggested that the MAOI activity in tobacco smoke may enhance a smoker’s mood (Pomerleau et al., 1984; Farzin and Mansouri, 2006; Smith et al., 2013; Choi et al., 2015) independently of their effects on dopamine reward from nicotine, and may contribute to smoker’s dependence on tobacco by this indirect route (Arnold et al., 2014). However, rather than a positive effect on mood, it is possible that MAO inhibition serves to intensify withdrawal symptoms (Malin et al., 2013). Relief of withdrawal symptoms then serves to improve mood. This potential interplay of influences is not yet fully understood.

The potential role for MAO inhibition in tobacco dependence has led to suggestions that MAO inhibitors could be used for smoking cessation (Biberman et al., 2003; George and Weinberger, 2008). Current consensus is that, after some promising preliminary results, the known MAO inhibitory drugs are not particularly useful for smoking cessation (Howes et al., 2020).

The proposed effects of MAO inhibitors on smokers are listed in Table 1.

## Other Components of Tobacco Smoke

Additional components of tobacco and tobacco smoke which have been associated with beneficial effects are listed in Table 2, below. They have been identified as a result of investigations of the active components supporting herbal use, but from plants other than tobacco. These compounds are all known components of tobacco (Rodgman and Perfetti, 2013), however, while their biological activity is of interest, their contribution to the overall effects of tobacco smoking is unknown.

**TABLE 2:** Additional tobacco smoke components with beneficial biological activity.

Component	Chemical structure	Observed effect	Application	References
Catechol	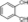	↓ amyloid-β fibril formation	Alzheimer’s disease	Huong et al., 2010
Hydroquinone	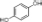	↓ a-synuclein fibrillation	Parkinson’s disease	Hong et al., 2009
Solanesol	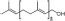	Neuroprotection	Stroke	Rajdev et al., 2020
Limonene	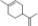	Neuroprotection	Alzheimer’s disease	Shin et al., 2020
Cembranoids	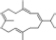	Nicotinic activation, neuroprotection	Alzheimer’s disease Parkinson’s disease	Ferchmin et al., 2009
Quercetin	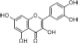	↓cognitive function ↓ amyloid-β fibril formation	Alzheimer’s disease Parkinson’s disease	Paula et al., 2019; Khan et al., 2019
Kaempferol	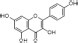	↑ striatal dopamine, SOD and GSH ↓ malondialdehyde	Parkinson’s disease	Li and Pu, 2011
Eugenol	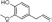	↓immobility in forced swim test	Depression	Irie et al., 2004
β-asarone	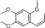	↑efficacy of memantine ↑cognitive deficit	Alzheimer’s disease	Han et al., 2020; Liu et al., 2016; Chang and Teng, 2018
Vanillin	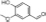	↓ oxidative stress response ↓behavioural impairment	Parkinson’s disease	Dhanalakshmi et al., 2016
Ferulic acid	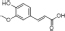	↑ serotonin and norepinephrine ↓immobility in forced swim test ↓brain capillary constriction	Depression Alzheimer’s disease	Chen et al., 2015; Wang et al., 2021
Caffeic acid	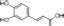	↓ immobility in forced swim test	Depression	Takeda et al., 2002
Chlorogenic acid	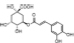	↓mitochondrial disfunction ↓ oxidative stress	Parkinson’s disease	Singh et al., 2020
Rutin	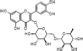	↓ immobility in tail suspension test ↓ effect of chronic induced stress	Depression, Anxiety	Parashar et al., 2017; Yusha’u et al., 2017
Naringenin	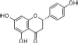	↓ amyloid-β toxicity ↓ immobility in tail suspension test ↑memory	Alzheimer’s disease Depression	Yi et al., 2012; Yang et al., 2014; Md et al., 2018
Naringin	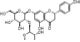	↓mitochondrial disfunction ↑memory	Alzheimer’s disease	Sachdeva et al., 2014
Scopoletin	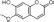	↓ anxiety-like behaviour	Anxiety	Luo et al., 2020
Esculetin	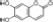	↓ immobility in forced swim test	Depression, Anxiety	Sulakhiya et al., 2016
Quinic acid	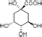	↓MAO-B, neuroprotection	Dementia	Liu et al., 2020

*SOD, superoxide dismutase; GSH, glutathione peroxidase.*

## Conclusion

It is accepted that nicotine has positive effects on cognition and attention in adults, even though having negative effects in infant and child development. For this reason, nicotine could be helpful in the management of a variety of mental health conditions and provide an explanation for high levels of smoking in disorders such as schizophrenia. Nicotine and its metabolite cotinine both stimulate cholinergic systems, which can be neuroprotective, and may also prevent ß-amyloid fibre aggregation in Alzheimer’s disease, and α-synuclein fibre formation in Parkinson’s disease. While the long-term overall effect of smoking is deleterious, the same may not be true for nicotine and cotinine. The therapeutic use of cotinine deserves further work, since it is expected to have a lower abuse potential than nicotine but may provide similar neuroprotective and cognitive effects.

The other most significant and potentially beneficial biological activity in tobacco smoke is monoamine oxidase inhibitory activity. This is likely to have an impact on the response to nicotine, and to increase the addictiveness of smoking, both by increasing the dopamine rewards from the nicotine, and by its effects (if any) on mood. The extent to which these effects on mood from smoking are due to relief of nicotine cravings, or directly caused by the modulation of monoamine oxidase enzyme activity in the brain remains uncertain. In this respect it is important to recall that the first generation of antidepressant drugs were selective MAO inhibitors, with some still currently used for treatment of major depressive disorders. This question will only be resolved when the causative agents of the MAO inhibition seen in smokers have been identified, and their effects can be studied independently of the effects of nicotine. It may prove possible to separate immediate MAO inhibitory activity from the long-term irreversible inhibitory factors and assess their effects separately once any irreversible inhibitors have been identified.

It is likely that MAO-B inhibitory activity in tobacco smoke, perhaps together with the combined neuroprotective effect of a variety of smoke components may be useful for symptom control in Parkinson’s disease, while MAO-A inhibitory activity is more likely to contribute to the alleviation of mood disorders.

While the contribution of biologically active molecules other than nicotine remains to be established it is clear that tobacco smoke contains many components which might have beneficial effects. While this in no way compensates for the overall deleterious effects of smoking, these effects may help explain the strength of tobacco dependence many smokers experience and are worthy of further study, so that we can disentangle the deleterious and beneficial effects, to better help smokers to stop smoking.

## Author Contributions

PT, RP, and PT-S guided the initial literature search, performed by SH. SH and PT wrote the manuscript. BE added expert advice. All authors contributed to the final shape of the manuscript.

## Conflict of Interest

The authors declare that the research was conducted in the absence of any commercial or financial relationships that could be construed as a potential conflict of interest.

## Publisher’s Note

All claims expressed in this article are solely those of the authors and do not necessarily represent those of their affiliated organizations, or those of the publisher, the editors and the reviewers. Any product that may be evaluated in this article, or claim that may be made by its manufacturer, is not guaranteed or endorsed by the publisher.

## References

[B1] Albuquerque E. X. Pereira E. F. Alkondon M. Rogers S. W. (2009). Mammalian nicotinic acetylcholine receptors: from structure to function. *Physiol. Rev.* 89 73–120. 10.1152/physrev.00015.2008 19126755 PMC2713585

[B2] Albuquerque E. X. Santos M. D. Alkondon M. Pereira E. F. Maelicke A. (2001). Modulation of nicotinic receptor activity in the central nervous system: a novel approach to the treatment of Alzheimer disease. *Alzheimer Dis. Assoc. Disord.* 15 S19–S25. 10.1097/00002093-200108001-00004 11669505

[B3] Alhowail A. H. (2021). Mechanisms underlying cognitive impairment induced by prenatal nicotine exposure: a literature review. *Eur. Rev. Med. Pharmacol. Sci.* 25 6057–6064. 10.26355/eurrev_202110_26884 34661266

[B4] Alkondon M. Pereira E. F. Almeida L. E. Randall W. R. Albuquerque E. X. (2000). Nicotine at concentrations found in cigarette smokers activates and desensitizes nicotinic acetylcholine receptors in CA1 interneurons of rat hippocampus. *Neuropharmacol* 39 2726–2739. 10.1016/s0028-3908(00)00156-8 11044743

[B5] Arnold M. M. Loughlin S. E. Belluzzi J. D. Leslie F. M. (2014). Reinforcing and neural activating effects of norharmane, a non-nicotine tobacco constituent, alone and in combination with nicotine. *Neuropharmacol* 85 293–304. 10.1016/j.neuropharm.2014.05.035 24907588

[B6] Aubin H. J. Rollema H. Svensson T. H. Winterer G. (2012). Smoking, quitting, and psychiatric disease: a review. *Neurosci. Biobehav. Rev.* 36 271–284. 10.1016/j.neubiorev.2011.06.007 21723317

[B7] Baler R. D. Volkow N. D. Fowler J. S. Benveniste H. (2008). Is fetal brain monoamine oxidase inhibition the missing link between maternal smoking and conduct disorders? *J. Psychiatr. Neurosci.* 33 187–195. 18592036 PMC2441882

[B8] Balfour D. J. (2009). The neuronal pathways mediating the behavioral and addictive properties of nicotine. *Handb. Exp. Pharmacol.* 192 209–233. 10.1007/978-3-540-69248-5_8 19184651

[B9] Belluzzi J. D. Wang R. Leslie F. M. (2005). Acetaldehyde enhances acquisition of nicotine self-administration in adolescent rats. *Neuropsychopharmacol* 30 705–712. 10.1038/sj.npp.1300586 15496937

[B10] Benowitz N. L. (1996). Pharmacology of nicotine: Addiction and therapeutics. *Annu. Rev. Pharmacol. Toxicol.* 36 597–613. 10.1146/annurev.pa.36.040196.003121 8725403

[B11] Benowitz N. L. (2009). Pharmacology of nicotine: addiction, smoking-induced disease, and therapeutics. *Annu. Rev. Pharmacol. Toxicol.* 49 57–71. 10.1146/annurev.pharmtox.48.113006.094742 18834313 PMC2946180

[B12] Benowitz N. L. (2010). Nicotine Addiction. *New England J. Med.* 362 2295–2303. 10.1056/NEJMra0809890 20554984 PMC2928221

[B13] Biberman R. Neumann R. Katzir I. Gerber Y. (2003). A randomized controlled trial of oral selegiline plus nicotine skin patch compared with placebo plus nicotine skin patch for smoking cessation. *Addiction* 98 1403–1407. 10.1046/j.1360-0443.2003.00524.x 14519177

[B14] Borroni E. Bohrmann B. Grueninger F. Prinssen E. Nave S. Loetscher H. (2017). Sembragiline: A Novel, Selective Monoamine Oxidase Type B Inhibitor for the Treatment of Alzheimer’s Disease. *J. Pharmacol. Exp. Ther.* 362 413–423. 10.1124/jpet.117.241653 28642233

[B15] Brennan K. A. Crowther A. Putt F. Roper V. Waterhouse U. Truman P. (2015). Tobacco particulate matter self-administration in rats: differential effects of tobacco type. *Addict Biol.* 20 227–235. 10.1111/adb.12099 24750334

[B16] Castagnoli K. Murugesan T. (2004). Tobacco leaf, smoke and smoking, MAO inhibitors, Parkinson’s disease and neuroprotection; are there links? *Neurotoxicol.* 25 279–291. 10.1016/S0161-813X(03)00107-4 14697903

[B17] Chan Y. L. Oliver B. G. Chen H. (2020). What lessons have we learnt about the impact of maternal cigarette smoking from animal models? *Clin. Exp. Pharmacol. Physiol.* 47 337–344. 10.1111/1440-1681.13182 31556137

[B18] Chang W. Teng J. (2018). Combined application of tenuigenin and beta-asarone improved the efficacy of memantine in treating moderate-to-severe Alzheimer’s disease. *Drug Des. Devel. Ther.* 12 455–462. 10.2147/DDDT.S155567 29551889 PMC5842771

[B19] Chen J. Lin D. Zhang C. Li G. Zhang N. Ruan L. (2015). Antidepressant-like effects of ferulic acid: involvement of serotonergic and norepinergic systems. *Metab. Brain Dis.* 30 129–136. 10.1007/s11011-014-9635-z 25483788

[B20] Chiamulera C. (2005). Cue reactivity in nicotine and tobacco dependence: a “multiple-action” model of nicotine as a primary reinforcement and as an enhancer of the effects of smoking-associated stimuli. *Brain Res. Rev.* 48 74–97. 10.1016/j.brainresrev.2004.08.005 15708629

[B21] Choi D. Ota S. Watanuki S. (2015). Does cigarette smoking relieve stress? Evidence from the event-related potential (ERP). *Int. J. Psychophysiol.* 98 470–476. 10.1016/j.ijpsycho.2015.10.005 26497442

[B22] Conley A. C. Key A. P. Taylor W. D. Albert K. M. Boyd B. D. Vega J. N. (2021). EEG as a Functional Marker of Nicotine Activity: Evidence From a Pilot Study of Adults With Late-Life Depression. *Front. Psychiatr.* 12:721874. 10.3389/fpsyt.2021.721874 35002791 PMC8732868

[B23] Costello M. R. Reynaga D. D. Mojica C. Y. Zaveri N. T. Belluzzi J. D. Leslie F. M. (2014). Comparison of the reinforcing properties of nicotine and cigarette smoke extract in rats. *Neuropsychopharmacol* 39 1843–1851. 10.1038/npp.2014.31 24513971 PMC4059892

[B24] Dhanalakshmi C. Janakiraman U. Manivasagam T. Justin Thenmozhi A. Essa M. M. Kalandar A. (2016). Vanillin Attenuated Behavioural Impairments, Neurochemical Deficts, Oxidative Stress and Apoptosis Against Rotenone Induced Rat Model of Parkinson’s Disease. *Neurochem. Res.* 41 1899–1910. 10.1007/s11064-016-1901-5 27038927

[B25] Di Chiara G. Imperato A. (1988). Drugs abused by humans preferentially increase synaptic dopamine concentrations in the mesolimbic system of freely moving rats. *Proc. Natl. Acad. Sci. U.S.A.* 85 5274–5278. 10.1073/pnas.85.14.5274 2899326 PMC281732

[B26] Dome P. Lazary J. Kalapos M. P. Rihmer Z. (2010). Smoking, nicotine and neuropsychiatric disorders. *Neurosci. Biobehav. Rev.* 34 295–342. 10.1016/j.neubiorev.2009.07.013 19665479

[B27] Esteves I. M. Lopes-Aguiar C. Rossignoli M. T. Ruggiero R. N. Broggini A. C. S. Bueno-Junior L. S. (2017). Chronic nicotine attenuates behavioral and synaptic plasticity impairments in a streptozotocin model of Alzheimer’s disease. *Neurosci* 353 87–97. 10.1016/j.neuroscience.2017.04.011 28433649

[B28] Farzin D. Mansouri N. (2006). Antidepressant-like effect of harmane and other beta-carbolines in the mouse forced swim test. *Eur. Neuropsychopharmacol.* 16 324–328. 10.1016/j.euroneuro.2005.08.005 16183262

[B29] Fekkes D. Bode W. T. (1993). Occurrence and partition of the β-carboline norharman in rat organs. *Life Sci.* 52 2045–2054. 10.1016/0024-3205(93)90689-Z 8502131

[B30] Ferchmin P. A. Pagan O. R. Ulrich H. Szeto A. C. Hann R. M. Eterovic V. A. (2009). Actions of octocoral and tobacco cembranoids on nicotinic receptors. *Toxicon* 54 1174–1182. 10.1016/j.toxicon.2009.02.033 19281835 PMC2783377

[B31] Fluharty M. Taylor A. E. Grabski M. Munafo M. R. (2017). The Association of Cigarette Smoking With Depression and Anxiety: A Systematic Review. *Nicotine. Tob. Res.* 19 3–13. 10.1093/ntr/ntw140 27199385 PMC5157710

[B32] Fowler J. S. Logan J. Wang G. J. Volkow N. D. (2003). Monoamine oxidase and cigarette smoking. *Neurotoxicol* 24 75–82. 10.1016/s0161-813x(02)00109-2 12564384

[B33] Fowler J. S. Volkow N. D. Wang G. J. Pappas N. Logan J. MacGregor R. (1996a). Inhibition of monoamine oxidase B in the brains of smokers. *Nature* 379 733–736. 10.1038/379733a0 8602220

[B34] Fowler J. S. Volkow N. D. Wang G. J. Pappas N. Logan J. Shea C. (1996b). Brain monoamine oxidase A inhibition in cigarette smokers. *Proc. Natl. Acad. Sci. U.S.A.* 93 14065–14069. 10.1073/pnas.93.24.14065 8943061 PMC19495

[B35] Gandelman J. A. Newhouse P. Taylor W. D. (2018). Nicotine and networks: Potential for enhancement of mood and cognition in late-life depression. *Neurosci. Biobehav. Rev.* 84 289–298. 10.1016/j.neubiorev.2017.08.018 28859996 PMC5729074

[B36] George T. P. Weinberger A. H. (2008). Monoamine oxidase inhibition for tobacco pharmacotherapy. *Clin. Pharmacol. Ther.* 83 619–621. 10.1038/sj.clpt.6100474 18091758 PMC3685473

[B37] Gigante A. F. Martino T. Iliceto G. Defazio G. (2017). Smoking and age-at-onset of both motor and non-motor symptoms in Parkinson’s disease. *Parkinsonism Relat. Disord.* 45 94–96. 10.1016/j.parkreldis.2017.09.022 28988683

[B38] Guillem K. Vouillac C. Azar M. R. Parsons L. H. Koob G. F. Cador M. (2005). Monoamine oxidase inhibition dramatically increases the motivation to self-administer nicotine in rats. *J. Neurosci.* 25 8593–8600. 10.1523/JNEUROSCI.2139-05.2005 16177026 PMC6725504

[B39] Hajdusianek W. Zorawik A. Waliszewska-Prosol M. Poreba R. Gac P. (2021). Tobacco and Nervous System Development and Function-New Findings 2015-2020. *Brain Sci.* 11:797. 10.3390/brainsci11060797 34208753 PMC8234722

[B40] Han Y. Wang N. Kang J. Fang Y. (2020). beta-Asarone improves learning and memory in Abeta1-42-induced Alzheimer’s disease rats by regulating PINK1-Parkin-mediated mitophagy. *Metab. Brain Dis.* 35 1109–1117. 10.1007/s11011-020-00587-2 32556928

[B41] Harris A. C. Mattson C. LeSage M. G. Keyler D. E. Pentel P. R. (2010). Comparison of the behavioral effects of cigarette smoke and pure nicotine in rats. *Pharmacol. Biochem. Behav.* 96 217–227. 10.1016/j.pbb.2010.05.008 20494826 PMC2887743

[B42] Harris A. C. Muelken P. LeSage M. G. (2020). beta-Carbolines found in cigarette smoke elevate intracranial self-stimulation thresholds in rats. *Pharmacol. Biochem. Behav.* 198:173041. 10.1016/j.pbb.2020.173041 32926882 PMC7554228

[B43] Harris A. C. Tally L. Muelken P. Banal A. Schmidt C. E. Cao Q. (2015). Effects of nicotine and minor tobacco alkaloids on intracranial-self-stimulation in rats. *Drug Alcohol Depend* 153 330–334. 10.1016/j.drugalcdep.2015.06.005 26094184 PMC4509975

[B44] Henningfield J. E. Fant R. V. (1999). Tobacco use as drug addiction: the scientific foundation. *NicotineTob. Res.* 1 S31–S35. 10.1080/14622299050011781 11768184

[B45] Herraiz T. Chaparro C. (2005). Human monoamine oxidase is inhibited by tobacco smoke: beta-carboline alkaloids act as potent and reversible inhibitors. *Biochem. Biophys. Res. Commun.* 326 378–386. 10.1016/j.bbrc.2004.11.033 15582589

[B46] Higuchi Y. Soga T. Parhar I. S. (2018). Potential Roles of microRNAs in the Regulation of Monoamine Oxidase A in the Brain. *Front. Mol. Neurosci.* 11:339. 10.3389/fnmol.2018.00339 30271325 PMC6149293

[B47] Hoek J. Gendall P. Gifford H. Pirikahu G. McCool J. Pene G. (2012). Tobacco branding, plain packaging, pictorial warnings, and symbolic consumption. *Qual. Health Res.* 22 630–639. 10.1177/1049732311431070 22203384

[B48] Hogg R. C. (2016). Contribution of Monoamine Oxidase Inhibition to Tobacco Dependence: A Review of the Evidence. *Nicotine Tob. Res.* 18 509–523. 10.1093/ntr/ntv245 26508396

[B49] Hong D. P. Fink A. L. Uversky V. N. (2009). Smoking and Parkinson’s disease: does nicotine affect alpha-synuclein fibrillation? *Biochim. Biophys. Acta* 1794 282–290. 10.1016/j.bbapap.2008.09.026 19013262 PMC2647853

[B50] Howes S. Hartmann-Boyce J. Livingstone-Banks J. Hong B. Lindson N. (2020). Antidepressants for smoking cessation. *Cochrane Database Syst. Rev.* 4:CD000031. 10.1002/14651858.CD000031.pub5 32319681 PMC7175455

[B51] Huang L. Z. Parameswaran N. Bordia T. Michael, McIntosh J. Quik M. (2009). Nicotine is neuroprotective when administered before but not after nigrostriatal damage in rats and monkeys. *J. Neurochem.* 109 826–837. 10.1111/j.1471-4159.2009.06011.x 19250334 PMC2677631

[B52] Hukkanen J. Jacob P. III Benowitz N. L. (2005). Metabolism and disposition kinetics of nicotine. *Pharmacol. Rev.* 57 79–115. 10.1124/pr.57.1.3 15734728

[B53] Huong V. T. Shimanouchi T. Shimauchi N. Yagi H. Umakoshi H. Goto Y. (2010). Catechol derivatives inhibit the fibril formation of amyloid-beta peptides. *J. Biosci. Bioeng.* 109 629–634. 10.1016/j.jbiosc.2009.11.010 20471605

[B54] Iarkov A. Mendoza C. Echeverria V. (2021). Cholinergic Receptor Modulation as a Target for Preventing Dementia in Parkinson’s Disease. *Front. Neurosci.* 15:665820. 10.3389/fnins.2021.665820 34616271 PMC8488354

[B55] Irie Y. Itokazu N. Anjiki N. Ishige A. Watanabe K. Keung W. M. (2004). Eugenol exhibits antidepressant-like activity in mice and induces expression of metallothionein-III in the hippocampus. *Brain Res.* 1011 243–246. 10.1016/j.brainres.2004.03.040 15157811

[B56] Jain A. (2003). Treating nicotine addiction. *BMJ* 327 1394–1395. 10.1136/bmj.327.7428.1394 14670889 PMC292997

[B57] Janes A. C. Zegel M. Ohashi K. Betts J. Molokotos E. Olson D. (2018). Nicotine normalizes cortico-striatal connectivity in non-smoking individuals with major depressive disorder. *Neuropsychopharmacol* 43 2445–2451. 10.1038/s41386-018-0069-x 29795403 PMC6180119

[B58] Kardani J. Sethi R. Roy I. (2017). Nicotine slows down oligomerisation of alpha-synuclein and ameliorates cytotoxicity in a yeast model of Parkinson’s disease. *Biochim. Biophys. Acta Mol. Basis Dis.* 1863 1454–1463. 10.1016/j.bbadis.2017.02.002 28167231

[B59] Khan H. Ullah H. Aschner M. Cheang W. S. Akkol E. K. (2019). Neuroprotective Effects of Quercetin in Alzheimer’s Disease. *Biomolecules* 10:59. 10.3390/biom10010059 31905923 PMC7023116

[B60] Kumari V. Gray J. A. ffytche D. H. Mitterschiffthaler M. T. Das M. Zachariah E. (2003). Cognitive effects of nicotine in humans: an fMRI study. *Neuroimage* 19 1002–1013. 10.1016/s1053-8119(03)00110-1 12880828

[B61] Launay J. M. Del Pino M. Chironi G. Callebert J. Peoc’h K. Megnien J. L. (2009). Smoking Induces Long-Lasting Effects through a Monoamine-Oxidase Epigenetic Regulation. *PLoS One* 4:e7959. 10.1371/journal.pone.0007959 19956754 PMC2775922

[B62] Laviolette S. R. van der Kooy D. (2004). The neurobiology of nicotine addiction: bridging the gap from molecules to behaviour. *Nat. Rev. Neurosci.* 5 55–65. 10.1038/nrn1298 14708004

[B63] Leonard S. Adler L. E. Benhammou K. Berger R. Breese C. R. Drebing C. (2001). Smoking and mental illness. *Pharmacol. Biochem. Behav.* 70 561–570. 10.1016/s0091-3057(01)00677-3 11796154

[B64] Lewis A. Miller J. H. Lea R. A. (2007). Monoamine oxidase and tobacco dependence. *Neurotoxicol* 28 182–195. 10.1016/j.neuro.2006.05.019 16859748

[B65] Li S. Pu X. P. (2011). Neuroprotective effect of kaempferol against a 1-methyl-4-phenyl-1,2,3,6-tetrahydropyridine-induced mouse model of Parkinson’s disease. *Biol. Pharm Bull.* 34 1291–1296. 10.1248/bpb.34.1291 21804220

[B66] Liu L. Liu Y. Zhao J. Xing X. Zhang C. Meng H. (2020). Neuroprotective Effects of D-(-)-Quinic Acid on Aluminum Chloride-Induced Dementia in Rats. *Evid. Based Complement Alternat. Med.* 2020:5602597. 10.1155/2020/5602597 32454864 PMC7240662

[B67] Liu S. J. Yang C. Zhang Y. Su R. Y. Chen J. L. Jiao M. M. (2016). Neuroprotective effect of beta-asarone against Alzheimer’s disease: regulation of synaptic plasticity by increased expression of SYP and GluR1. *Drug Des. Devel. Ther.* 10 1461–1469. 10.2147/DDDT.S93559 27143853 PMC4841421

[B68] Lopez-Arrieta J. M. Rodriguez J. L. Sanz F. (2001). Efficacy and safety of nicotine on Alzheimer’s disease patients. *Cochrane Database Syst. Rev.* 2001:CD001749. 10.1002/14651858.CD001749 11406005

[B69] Luo L. Sun T. Yang L. Liu A. Liu Q. Q. Tian Q. Q. (2020). Scopoletin ameliorates anxiety-like behaviors in complete Freund’s adjuvant-induced mouse model. *Mol. Brain* 13:15. 10.1186/s13041-020-0560-2 32019580 PMC7001522

[B70] Majdi A. Sadigh-Eteghad S. Gjedde A. (2021). Effects of transdermal nicotine delivery on cognitive outcomes: A meta-analysis. *Acta Neurol. Scand.* 144 179–191. 10.1111/ane.13436 33899218

[B71] Malin D. H. Moon W. D. Goyarzu P. Barclay E. Magallanes N. Vela A. J. (2013). Inhibition of monoamine oxidase isoforms modulates nicotine withdrawal syndrome in the rat. *Life Sci.* 93 448–453. 10.1016/j.lfs.2013.08.006 23988853

[B72] Marusich J. A. Darna M. Wilson A. G. Denehy E. D. Ebben A. Deaciuc A. G. (2017). Tobacco’s minor alkaloids: Effects on place conditioning and nucleus accumbens dopamine release in adult and adolescent rats. *Eur. J. Pharmacol.* 814 196–206. 10.1016/j.ejphar.2017.08.029 28844873 PMC6563910

[B73] Md S. Gan S. Y. Haw Y. H. Ho C. L. Wong S. Choudhury H. (2018). In vitro neuroprotective effects of naringenin nanoemulsion against beta-amyloid toxicity through the regulation of amyloidogenesis and tau phosphorylation. *Int. J. Biol. Macromol.* 118 1211–1219. 10.1016/j.ijbiomac.2018.06.190 30001606

[B74] Mendez-Alvarez E. Soto-Otero R. Sanchez-Sellero I. Lopez-Rivadulla Lamas M. (1997). Inhibition of brain monoamine oxidase by adducts of 1,2,3,4-tetrahydroisoquinoline with components of cigarette smoke. *Life Sci.* 60 1719–1727. 10.1016/s0024-3205(97)00114-8 9129127

[B75] Mihailescu S. Drucker-Colin R. (2000). Nicotine, brain nicotinic receptors, and neuropsychiatric disorders. *Arch. Med. Res.* 31 131–144. 10.1016/s0188-4409(99)00087-9 10880717

[B76] Molas S. DeGroot S. R. Zhao-Shea R. Tapper A. R. (2017). Anxiety and Nicotine Dependence: Emerging Role of the Habenulo-Interpeduncular Axis. *Trends Pharmacol. Sci.* 38 169–180. 10.1016/j.tips.2016.11.001 27890353 PMC5258775

[B77] Moolchan E. T. Aung A. T. Henningfield J. E. (2003). Treatment of adolescent tobacco smokers: issues and opportunities for exposure reduction approaches. *Drug Alcohol Depend* 70 223–232. 10.1016/s0376-8716(03)00012-7 12757960

[B78] Moylan S. Jacka F. N. Pasco J. A. Berk M. (2013). How cigarette smoking may increase the risk of anxiety symptoms and anxiety disorders: a critical review of biological pathways. *Brain Behav.* 3 302–326. 10.1002/brb3.137 23785661 PMC3683289

[B79] Naoi M. Maruyama W. Nagy G. M. (2004). Dopamine-derived salsolinol derivatives as endogenous monoamine oxidase inhibitors: occurrence, metabolism and function in human brains. *Neurotoxicol* 25 193–204. 10.1016/S0161-813X(03)00099-8 14697894

[B80] Newhouse P. Singh A. Potter A. (2004a). Nicotine and nicotinic receptor involvement in neuropsychiatric disorders. *Curr. Top. Med. Chem.* 4 267–282. 10.2174/1568026043451401 14754447

[B81] Newhouse P. A. Potter A. Singh A. (2004b). Effects of nicotinic stimulation on cognitive performance. *Curr. Opin. Pharmacol.* 4 36–46. 10.1016/j.coph.2003.11.001 15018837

[B82] Nop O. Senft Miller A. Culver H. Makarewicz J. Dumas J. A. (2021). Nicotine and Cognition in Cognitively Normal Older Adults. *Front. Aging Neurosci.* 13:640674. 10.3389/fnagi.2021.640674 34025390 PMC8131527

[B83] Parashar A. Mehta V. Udayabanu M. (2017). Rutin alleviates chronic unpredictable stress-induced behavioral alterations and hippocampal damage in mice. *Neurosci. Lett.* 656 65–71. 10.1016/j.neulet.2017.04.058 28732760

[B84] Patsenka A. Antkiewicz-Michaluk L. (2004). Inhibition of rodent brain monoamine oxidase and tyrosine hydroxylase by endogenous compounds - 1,2,3,4-tetrahydro-isoquinoline alkaloids. *Pol. J. Pharmacol.* 56 727–734. 15662085

[B85] Paula P. C. Angelica Maria S. G. Luis C. H. Gloria Patricia C. G. (2019). Preventive Effect of Quercetin in a Triple Transgenic Alzheimer’s Disease Mice Model. *Molecules* 24:2287. 10.3390/molecules24122287 31226738 PMC6630340

[B86] Pomerleau O. F. Turk D. C. Fertig J. B. (1984). The effects of cigarette smoking on pain and anxiety. *Addict Behav.* 9 265–271. 10.1016/0306-4603(84)90018-2 6496202

[B87] Postma P. Gray J. A. Sharma T. Geyer M. Mehrotra R. Das M. (2006). A behavioural and functional neuroimaging investigation into the effects of nicotine on sensorimotor gating in healthy subjects and persons with schizophrenia. *Psychopharmacol* 184 589–599. 10.1007/s00213-006-0307-5 16456657

[B88] Quertemont E. Tambour S. (2004). Is ethanol a pro-drug? The role of acetaldehyde in the central effects of ethanol. *Trends Pharmacol. Sci.* 25 130–134. 10.1016/j.tips.2004.01.00 15019267

[B89] Rajdev K. Siddiqui E. M. Jadaun K. S. Mehan S. (2020). Neuroprotective potential of solanesol in a combined model of intracerebral and intraventricular hemorrhage in rats. *IBRO Rep.* 8 101–114. 10.1016/j.ibror.2020.03.001 32368686 PMC7184235

[B90] Riveles K. Huang L. Z. Quik M. (2008). Cigarette smoke, nicotine and cotinine protect against 6-hydroxydopamine-induced toxicity in SH-SY5Y cells. *Neurotoxicol* 29 421–427. 10.1016/j.neuro.2008.02.001 18359086 PMC2486261

[B91] Rodgman A. Perfetti T. A. (2013). *The Chemical Components of Tobacco and Tobacco Smoke*, 2nd Edn. Boca Raton: CRC Press. 10.1201/b13973

[B92] Rommelspacher H. Meier-Henco M. Smolka M. Kloft C. (2002). The levels of norharman are high enough after smoking to affect monoamineoxidase B in platelets. *Eur. J. Pharmacol.* 441 115–125. 10.1016/s0014-2999(02)01452-8 12007928

[B93] Rose J. E. (2006). Nicotine and nonnicotine factors in cigarette addiction. *Psychopharmacol* 184 274–285. 10.1007/s00213-005-0250-x 16362402

[B94] Sacco K. A. Bannon K. L. George T. P. (2004). Nicotinic receptor mechanisms and cognition in normal states and neuropsychiatric disorders. *J. Psychopharmacol.* 18 457–474. 10.1177/026988110401800403 15582913 PMC1201375

[B95] Sachdeva A. K. Kuhad A. Chopra K. (2014). Naringin ameliorates memory deficits in experimental paradigm of Alzheimer’s disease by attenuating mitochondrial dysfunction. *Pharmacol. Biochem. Behav.* 127 101–110. 10.1016/j.pbb.2014.11.002 25449356

[B96] Sari Y. Khalil A. (2015). Monoamine Oxidase Inhibitors Extracted from Tobacco Smoke as Neuroprotective Factors for Potential Treatment of Parkinson’s Disease. *CNS Neurol. Disord. Drug Targets* 14 777–785. 10.2174/1871527314666150325235608 25808895

[B97] Scott J. G. Matuschka L. Niemela S. Miettunen J. Emmerson B. Mustonen A. (2018). Evidence of a Causal Relationship Between Smoking Tobacco and Schizophrenia Spectrum Disorders. *Front. Psychiatr.* 9:607. 10.3389/fpsyt.2018.00607 30515111 PMC6255982

[B98] Seeman J. I. Dixon M. Haussmann H.-J. (2002). Acetaldehyde in mainstream tobacco smoke: formation and occurrence in smoke and bioavailability in the smoker. *Chem. Res. Toxicol.* 15 1331–1350. 10.1021/tx020069f 12437324

[B99] Sharama S. (2016). *Monoamine Oxidase Inhibitors : clinical Pharmacology, Benefits, and Potential Health Risks.* New York: Nova Science Publishers Inc.

[B100] Shin M. Liu Q. F. Choi B. Shin C. Lee B. Yuan C. (2020). Neuroprotective Effects of Limonene (+) against Abeta42-Induced Neurotoxicity in a Drosophila Model of Alzheimer’s Disease. *Biol. Pharm. Bull.* 43 409–417. 10.1248/bpb.b19-00495 31875578

[B101] Singh S. S. Rai S. N. Birla H. Zahra W. Rathore A. S. Dilnashin H. (2020). Neuroprotective Effect of Chlorogenic Acid on Mitochondrial Dysfunction-Mediated Apoptotic Death of DA Neurons in a Parkinsonian Mouse Model. *Oxid Med. Cell Longev.* 2020:6571484. 10.1155/2020/6571484 32566093 PMC7273475

[B102] Smith D. M. Fisher D. Blier P. Ilivitsky V. Knott V. (2016a). The separate and combined effects of monoamine oxidase A inhibition and nicotine on resting state EEG. *J. Psychopharmacol.* 30 56–62. 10.1177/0269881115613518 26537155

[B103] Smith T. T. Rupprecht L. E. Cwalina S. N. Onimus M. J. Murphy S. E. Donny E. C. (2016b). Effects of Monoamine Oxidase Inhibition on the Reinforcing Properties of Low-Dose Nicotine. *Neuropsychopharmacol* 41 2335–2343. 10.1038/npp.2016.36 26955970 PMC4946064

[B104] Smith K. L. Ford G. K. Jessop D. S. Finn D. P. (2013). Behavioural, neurochemical and neuroendocrine effects of the endogenous beta-carboline harmane in fear-conditioned rats. *J. Psychopharmacol* 27 162–170. 10.1177/0269881112460108 23015542

[B105] Smith T. T. Schaff M. B. Rupprecht L. E. Schassburger R. L. Buffalari D. M. Murphy S. E. (2015). Effects of MAO inhibition and a combination of minor alkaloids, beta-carbolines, and acetaldehyde on nicotine self-administration in adult male rats. *Drug Alcohol Depend* 155 243–252. 10.1016/j.drugalcdep.2015.07.002 26257022 PMC4581969

[B106] Sulakhiya K. Keshavlal G. P. Bezbaruah B. B. Dwivedi S. Gurjar S. S. Munde N. (2016). Lipopolysaccharide induced anxiety- and depressive-like behaviour in mice are prevented by chronic pre-treatment of esculetin. *Neurosci. Lett.* 611 106–111. 10.1016/j.neulet.2015.11.031 26620836

[B107] Syrjanen K. Eronen K. Hendolin P. Paloheimo L. Eklund C. Backstrom A. (2017). Slow-release L-Cysteine (Acetium(R)) Lozenge Is an Effective New Method in Smoking Cessation. A Randomized, Double-blind, Placebo-controlled Intervention. *Anticancer Res.* 37 3639–3648. 10.21873/anticanres.11734 28668855

[B108] Takeda H. Tsuji M. Inazu M. Egashira T. Matsumiya T. (2002). Rosmarinic acid and caffeic acid produce antidepressive-like effect in the forced swimming test in mice. *Eur. J. Pharmacol.* 449 261–267. 10.1016/s0014-2999(02)02037-x 12167468

[B109] Talhout R. Opperhuizen A. van Amsterdam J. G. (2007). Role of acetaldehyde in tobacco smoke addiction. *Eur. Neuropsychopharmacol.* 17 627–636. 10.1016/j.euroneuro.2007.02.013 17382522

[B110] Tan X. Ingraham C. M. McBride W. J. Ding Z. M. (2022). The involvement of mesolimbic dopamine system in cotinine self-administration in rats. *Behav. Brain Res.* 417:113596. 10.1016/j.bbr.2021.113596 34562552 PMC8578415

[B111] Tan X. Vrana K. Ding Z. M. (2021). Cotinine: Pharmacologically Active Metabolite of Nicotine and Neural Mechanisms for Its Actions. *Front. Behav. Neurosci.* 15:758252. 10.3389/fnbeh.2021.758252 34744656 PMC8568040

[B112] Terry A. V. Jr. Hernandez C. M. Hohnadel E. J. Bouchard K. P. Buccafusco J. J. (2005). Cotinine. A Neuroactive Metabolite of Nicotine: Potential for Treating Disorders of Impaired Cognition. *CNS Drug Rev.* 11 229–252. 10.1111/j.1527-3458.2005.tb00045.x 16389292 PMC6741756

[B113] Thiriez C. Villafane G. Grapin F. Fenelon G. Remy P. Cesaro P. (2011). Can nicotine be used medicinally in Parkinson’s disease? *Expert. Rev. Clin. Pharmacol.* 4 429–436. 10.1586/ecp.11.27 22114853

[B114] Truman P. Grounds P. Brennan K. A. (2017). Monoamine oxidase inhibitory activity in tobacco particulate matter: Are harman and norharman the only physiologically relevant inhibitors? *Neurotoxicol* 59 22–26. 10.1016/j.neuro.2016.12.010 28057462

[B115] U.S. Department of Health and Human Services. (2004). *The Health Consequences of Smoking: A Report of the Surgeon General.* Atlanta, GA: U.S. Department of Health and Human Services

[B116] Veljkovic E. Xia W. Phillips B. Wong E. T. Ho J. Oviedo A. (2018). “Parkinson’s Disease,” in *Nicotine and Other Tobacco Compounds in Neurodegenerative and Psychiatric Diseases. Part 1: Overview of Epidemiological Data on Smoking and Preclinical and Clinical Data on Nicotine*, Chap. 1, eds Veljkovic E. Xia W. Phillips B. Wong E. T. Ho J. Oviedo A. (Cambridge: Academic Press). 10.1016/B978-0-12-812922-7.00001-9

[B117] Venkatakrishnan P. Gairola C. G. Castagnoli N. Jr. Miller R. T. (2009). Naphthoquinones and bioactive compounds from tobacco as modulators of neuronal nitric oxide synthase activity. *Phytother. Res.* 23 1663–1672. 10.1002/ptr.2789 19367663 PMC2788052

[B118] Villafane G. Thiriez C. Audureau E. Straczek C. Kerschen P. Cormier-Dequaire F. (2018). High-dose transdermal nicotine in Parkinson’s disease patients: a randomized, open-label, blinded-endpoint evaluation phase 2 study. *Eur. J. Neurol.* 25 120–127. 10.1111/ene.13474 28960663

[B119] Villegier A. S. Belluzzi J. D. Leslie F. M. (2011). Serotonergic mechanism underlying tranylcypromine enhancement of nicotine self-administration. *Synapse* 65 479–489. 10.1002/syn.20864 20936688 PMC3070204

[B120] Villegier A. S. Salomon L. Granon S. Changeux J. P. Belluzzi J. D. Leslie F. M. (2006). Monoamine oxidase inhibitors allow locomotor and rewarding responses to nicotine. *Neuropsychopharmacol* 31 1704–1713. 10.1038/sj.npp.1300987 16395299

[B121] Wang N. Y. Li J. N. Liu W. L. Huang Q. Li W. X. Tan Y. H. (2021). Ferulic Acid Ameliorates Alzheimer’s Disease-like Pathology and Repairs Cognitive Decline by Preventing Capillary Hypofunction in APP/PS1 Mice. *Neurotherapeutics* 18 1064–1080. 10.1007/s13311-021-01024-7 33786807 PMC8423929

[B122] Waterhouse U. Brennan K. A. Ellenbroek B. A. (2018). Nicotine self-administration reverses cognitive deficits in a rat model for schizophrenia. *Addict Biol.* 23 620–630. 10.1111/adb.12517 28497655

[B123] West R. (2017). Tobacco smoking: Health impact, prevalence, correlates and interventions. *Psychol. Health* 32 1018–1036. 10.1080/08870446.2017.1325890 28553727 PMC5490618

[B124] West R. Cox S. (2021). The 1988 US Surgeon General’s report Nicotine Addiction: how well has it stood up to three more decades of research? *Addiction* [Epub Online ahead of print]. 10.1111/add.15754 34817099

[B125] Wu J. Lukas R. J. (2011). Naturally-expressed nicotinic acetylcholine receptor subtypes. *Biochem. Pharmacol.* 82 800–807. 10.1016/j.bcp.2011.07.067 21787755 PMC3163308

[B126] Yang W. Ma J. Liu Z. Lu Y. Hu B. Yu H. (2014). Effect of naringenin on brain insulin signaling and cognitive functions in ICV-STZ induced dementia model of rats. *Neurol. Sci.* 35 741–751. 10.1007/s10072-013-1594-3 24337945

[B127] Yi L. T. Li J. Li H. C. Su D. X. Quan X. B. He X. C. (2012). Antidepressant-like behavioral, neurochemical and neuroendocrine effects of naringenin in the mouse repeated tail suspension test. *Prog. Neuropsychopharmacol. Biol. Psychiatr.* 39 175–181. 10.1016/j.pnpbp.2012.06.009 22709719

[B128] Yu P. H. Boulton A. A. (1987). Irreversible inhibition of monoamine oxidase by some components of cigarette smoke. *Life Sci.* 41 675–682. 10.1016/0024-3205(87)90446-2 3613836

[B129] Yusha’u Y. Muhammad U. A. Nze M. Egwuma J. M. Igomu O. J. Abdulkadir M. (2017). Modulatory Role of Rutin Supplement on Open Space Forced Swim Test Murine Model of Depression. *Niger J. Physiol. Sci.* 32 201–205. 29485642

[B130] Zhang J. Liu Q. Chen Q. Liu N. Q. Li F. L. Lu Z. B. (2006). Nicotine attenuates beta-amyloid-induced neurotoxicity by regulating metal homeostasis. *FASEB J.* 20 1212–1214. 10.1096/fj.05-5214fje 16627626

